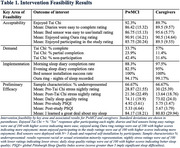# The feasibility of a tai chi for sleep intervention for persons with MCI and their caregivers

**DOI:** 10.1002/alz70858_102407

**Published:** 2025-12-26

**Authors:** Emily L. Giannotto, Amy D. Rodriguez, Jennifer DuBose, Agata Rozga, Gari Clifford, Clementine Rasheed, Tracy Moon, Muslimah LaForce, Allan I. Levey

**Affiliations:** ^1^ Emory University School of Medicine, Atlanta, GA, USA; ^2^ Georgia Institute of Technology, Atlanta, GA, USA; ^3^ Emory University, Atlanta, GA, USA

## Abstract

**Background:**

Persons with mild cognitive impairment (PwMCI) and caregivers commonly experience low quality sleep which is associated with increased stress. Tai Chi has shown tremendous promise for stress reduction and improving sleep quality in older adults and those with MCI. Our objective was to test the feasibility of a Tai Chi for sleep intervention among PwMCI and their caregivers.

**Method:**

Participants were 12 PwMCI (3 female, 50% African American/Black, M = 75.4 years) diagnosed from Emory's Cognitive Neurology Clinic and their 12 spousal caregivers (8 female, 50% African American/Black, M = 74.6 years). Participants used a bed sensor, an Oura ring, and completed morning and evening sleep diaries for 10 days. The evening diary included an embedded 10‐minute Tai Chi video curated by a Tai Chi master. Data were analyzed using descriptive statistics (see Table 1) and paired t‐tests to assess feasibility according to acceptability, demand, implementation, and preliminary efficacy.

**Result:**

*Acceptability*: Participants reacted positively to each of the intervention components and the study overall. Participants enjoyed Tai Chi, the bed sensors were easy to deploy, participants enjoyed using the Oura ring, and post‐participation study enjoyment ratings were high.

*Demand*: High interest in study participation among PwMCI and caregivers was supported by greater than 55% of nightly Tai Chi participation. However, caregivers were more likely than PwMCI to complete Tai Chi.

*Implementation*: The study components were largely executed as planned. Diary completion rates across the morning and evening forms were very high for PwMCI (85.83%) and caregivers (96.25%). The bed sensors were successfully self‐installed for five dyads and Oura ring use was near‐perfect across all participants.

*Preliminary efficacy*: Although limited, sleep and stress data from our diverse convenience sample supports employing our intervention approach and methods in a larger study focused on quantifying and improving sleep for PwMCI and their caregivers. Nightly stress was lower on average after completing Tai Chi compared to before for PwMCI and was significantly lower for caregivers (t(74) = 5.08, *p* = < .001).

**Conclusion:**

Our Tai Chi for sleep intervention was feasible for PwMCI and their caregivers. This approach warrants further exploration for improving sleep quality and stress reduction.